# Even short-term training improves the skills of novice exoscope users: a prospective laboratory experiment

**DOI:** 10.1007/s00701-024-05975-6

**Published:** 2024-03-01

**Authors:** Ville Vasankari, Ahmad Hafez, Anni Pohjola, Anna Maria Auricchio, Francesco Calvanese, Tobias Rossmann, Michael Veldeman, Ines Badic, Eliisa Netti, Ilari Rautalin, Ville Nurminen, Rahul Raj, Mika Niemelä, Martin Lehecka

**Affiliations:** 1 Department of Neurosurgery, Helsinki University Hospital and University of Helsinki, P.O. Box 266, 00029 Helsinki, Finland; 2 Department of Neurosurgery, Fondazione Policlinico Universitario A. Gemelli IRCCS, Università Cattolica del Sacro Cuore, Rome, Italy; 3 Department of Neurosurgery, Neuromed Campus, Kepler University Hospital, Johannes Kepler University, Linz, Austria; 4 Department of Neurosurgery, RWTH Aachen University Hospital, Aachen, Germany; 5 Sigmund Freud University Vienna, Vienna, Austria; 6 National Institute for Stroke and Applied Neurosciences, Auckland University of Technology, Auckland, New Zealand

**Keywords:** Bypass, Exoscope, Microscope, Microvascular anastomosis, Neurosurgery, Suturing

## Abstract

**Background:**

The surgical 3D exoscopes have recently been introduced as an alternative to the surgical microscopes in microneurosurgery. Since the exoscope availability is still limited, it is relevant to know whether even a short-term exoscope training develops the skills needed for performing exoscope-assisted surgeries.

**Methods:**

Ten participants (six consultants, four residents) performed two laboratory bypass test tasks with a 3D exoscope (Aesculap Aeos®). Six training sessions (6 h) were performed in between (interval of 2–5 weeks) on artificial models. The participants were divided into two groups: test group (*n* = 6) trained with the exoscope and control group (*n* = 4) with a surgical microscope. The test task was an artificial end-to-side microsurgical anastomosis model, using 12 interrupted 9–0 sutures and recorded on video. We compared the individual as well as group performance among the test subjects based on suturing time, anastomosis quality, and manual dexterity.

**Results:**

Altogether, 20 bypass tasks were performed (baseline *n* = 10, follow-up *n* = 10). The median duration decreased by 28 min and 44% in the exoscope training group. The decrease was steeper (29 min, 45%) among the participants with less than 6 years of microneurosurgery experience compared to the more experienced participants (13 min, 24%). After training, the participants with at least 1-year experience of using the exoscope did not improve their task duration. The training with the exoscope led to a greater time reduction than the training with the microscope (44% *vs* 17%).

**Conclusions:**

Even short-term training with the exoscope led to marked improvements in exoscope-assisted bypass suturing among novice microneurosurgeons. For the more experienced participants, a plateau in the initial learning curve was reached quickly. A much longer-term effort might be needed to witness further improvement in this user group.

**Supplementary Information:**

The online version contains supplementary material available at 10.1007/s00701-024-05975-6.

## Introduction

In neurosurgery, the operating microscope represents the gold standard for magnification and illumination of the surgical field. Recently, 3D exoscopes have been introduced as an alternative to the surgical microscope [6]. Several benefits of exoscope-assisted surgery, such as improved ergonomics and higher magnification, have been reported in previous studies, mostly performed in experimental bypass models and cadavers [2, 3, 5]. The exoscope has been shown to produce non-inferior clinical results even in complex neurosurgical procedures such as aneurysm surgery or skull base tumors [9, 11]. However, some limitations have also been mentioned, such as inferior illumination in deep locations and longer duration of surgery in highly challenging tasks [3, 5, 7].

Since the exoscope availability is limited in most neurosurgical units, it is relevant to know whether short-term exoscope training develops the skills needed for performing exoscope-assisted surgery. Furthermore, it is relevant to assess how previous microsurgical experience affect the adaptation process [10].

We designed an experimental laboratory study to evaluate the effect of short-term exoscope practice using an artificial end-to-side microanastomosis model. Our hypotheses were (1) less experienced microsurgeons will have a steeper learning-curve than the more experienced ones and (2) training with the exoscope will improve work under exoscope more than training with a microscope and (3) microsurgical training as such and adaptation to the test task will still have a positive effect, and (4) there is a limit to how much one can improve in a small number of training sessions.

## Methods and materials

### Participants

We included ten participants (three women, seven men) with a median of 6 years (range 0.5–10.0) of neurosurgical training. All participants performed two bypass test tasks and six training sessions in between. Data collection was conducted between December 2022 and January 2023. All participants were included as study authors and gave their informed consent to participation. Therefore, ethical approval was not required.

### Study design

All participants performed the same experimental end-to-side anastomosis task at two time points: at the baseline and after 6 h of dedicated microsurgical training (follow-up). The follow-up task was conducted within 2 to 5 weeks after completing the baseline task. Six separate 1-h training sessions were organized for each participant between the baseline and the follow-up tasks. The training sessions consisted of microsurgical bypass training with synthetic bypass tubes but not the actual test task.

The participants were divided into two groups: (i) an exoscope training group (i.e., test group) and (ii) a microscope training group (i.e., control group). The previous microsurgical experience of the participants was considered to achieve balanced groups. The exoscope training group included four consultant neurosurgeons and two residents, and the microscope training group included two consultant neurosurgeons and two residents. Two of the consultant neurosurgeons in both groups had at least 1-year experience using the exoscope, whereas none of the residents had operated with an exoscope prior. Some of the surgeons with the most experience had previously performed actual surgeries with the exoscope, positioning them in a different phase on the learning curve. We divided these surgeons into the two training groups in order to minimize the effect of previous exoscope expertise. We did not control the use of exoscope in the surgeries that they performed during the data collection. However, the surgeons involved in the study did not perform bypass surgeries prior or during the data collection. Three participants in the exoscope group had performed 15–30 endoscopic surgeries as main surgeon prior the data collection. None of the participants in the microscope group had previous endoscopic experience. Each group trained only with either the microscope or the exoscope, but baseline and follow-up tasks were performed with the exoscope by all participants.

### Standardized end-to-side bypass model

**Fig. 1:**
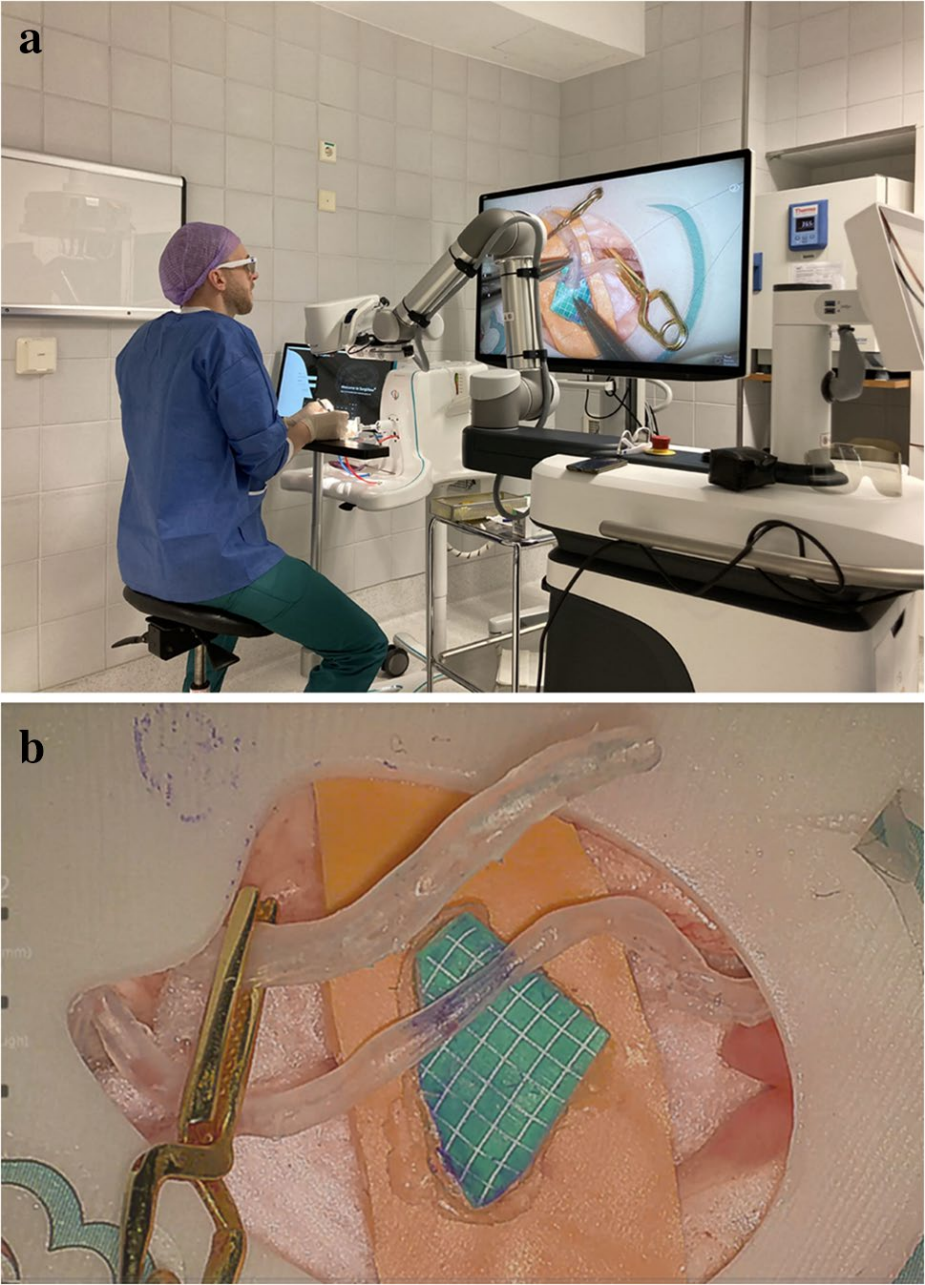
**a** The setup for the exoscope-assisted end-to-side bypass task. **b** Exoscope view of the end-to-side anastomosis model (SurgTrain.™ 4D Neurosurgical Simulator, SurgeonsLab)

A commercial microsurgical end-to-side bypass model (SurgeonsLab®, SurgTrain™ 4D Neurosurgical Simulator, Bern, Switzerland) was used for the microsurgical tasks at baseline and follow-up. The bypass model was created using 3D-printed synthetic tubes with diameter of 2.5–3 mm in the depth of 10 mm from the surface (Fig. 1). The tubes were positioned to simulate a real superficial temporal artery (STA) to cortical middle cerebral artery (MCA) bypass surgery.

The microsurgical bypass tasks (baseline and follow-up) were performed using a 3D digital exoscope (Aesculap Aeos ®) controlled with a foot pedal in sitting or standing position. A training session on how to use the exoscope was organized for the participants without previous exoscope experience. The setup for the anastomosis task is shown in Fig. 1. The task consisted of (a) applying a temporary clip to the donor vessel, (b) performing an arteriotomy to the donor vessel, (c) highlighting the anastomosis site on the recipient vessel with a marker, (d) applying two temporary clips to the recipient vessel, (e) performing an arteriotomy to the recipient, (f) attaching the donor to the recipient vessel with 12 interrupted 9–0 sutures (Dafilon®, B. Braun, Melsungen, Germany), and (g) removing the temporary clips.

All participants used the same set of microinstruments in the baseline and follow-up tasks, including two jewelers’ forceps, a microneedle holder and microscissors. The duration of the task was defined as the time from applying the first temporary clip to the recipient vessel to removing the last temporary clip (theoretical ischemia time). The task was terminated if the participant could not complete it in 75 min. All tasks were video-recorded, and completed anastomoses were stored for further inspection. The recordings were analyzed for three parameters: (1) how many times both instruments were out of exoscope’s view, (2) how many times the needle was accidentally dropped inside or outside the model (not including when the needle was laid down for tying the suture), and (3) how many times the exoscope image was out of focus. Due to technical problems, one recording of the baseline task (microscope group) and one recording of the follow-up task (exoscope group) were not available. The microanastomosis was assigned 0–2 points based on its quality. One point was granted for equally distributed sutures (less than 2:1 deviation in the distance between any adjacent sutures) and one point if the anastomosis orifice was larger than the recipient’s cross-sectional plane [4]. These quality parameters were selected as they are clinically relevant for eventual functionality (adequate flow without leakage) of a vascular anastomosis. Flow itself was not measured since unlike in biological tissues, there was too much leakage between the anastomotic ends of the plastic tube material to allow for any meaningful measurements. Of all the parameters monitored during and after the task, the participants were only aware of the time when performing the task.

Video 1**.** The exoscope-assisted, experimental end-to-side bypass task after six hours of training.

### Statistical analysis

We present time data as medians with ranges in minutes and seconds. We compared the baseline, the follow-up, and time improvement between the exoscope and microscope training groups using a Wilcoxon Rank Sum Test. For additional analysis, the participants were divided into two groups based on their previous experience in microsurgery: (a) less experienced ones (< 6 years of microsurgical training) and (b) more experienced ones (≥ 6 years of microsurgical training). Further analysis was also based on previous exoscopic experience: (i) novice exoscope users (< 1 year of experience) and (ii) more experienced exoscope users (≥ 1 year of experience).

## Results

### Duration of the test task

The ten participants performed one baseline and one follow-up task each (total of 20 exoscope-assisted bypasses). All anastomoses were completed with 12 sutures. At the baseline, the median task duration was 47 min (range 29–73 min). Finishing the task took longer among participants in the exoscope training group compared to the microscope training group (median 63 min *vs* 34 min, respectively). The exoscope training group included more novice exoscope users (67% *vs* 50%).

**Fig. 2:**
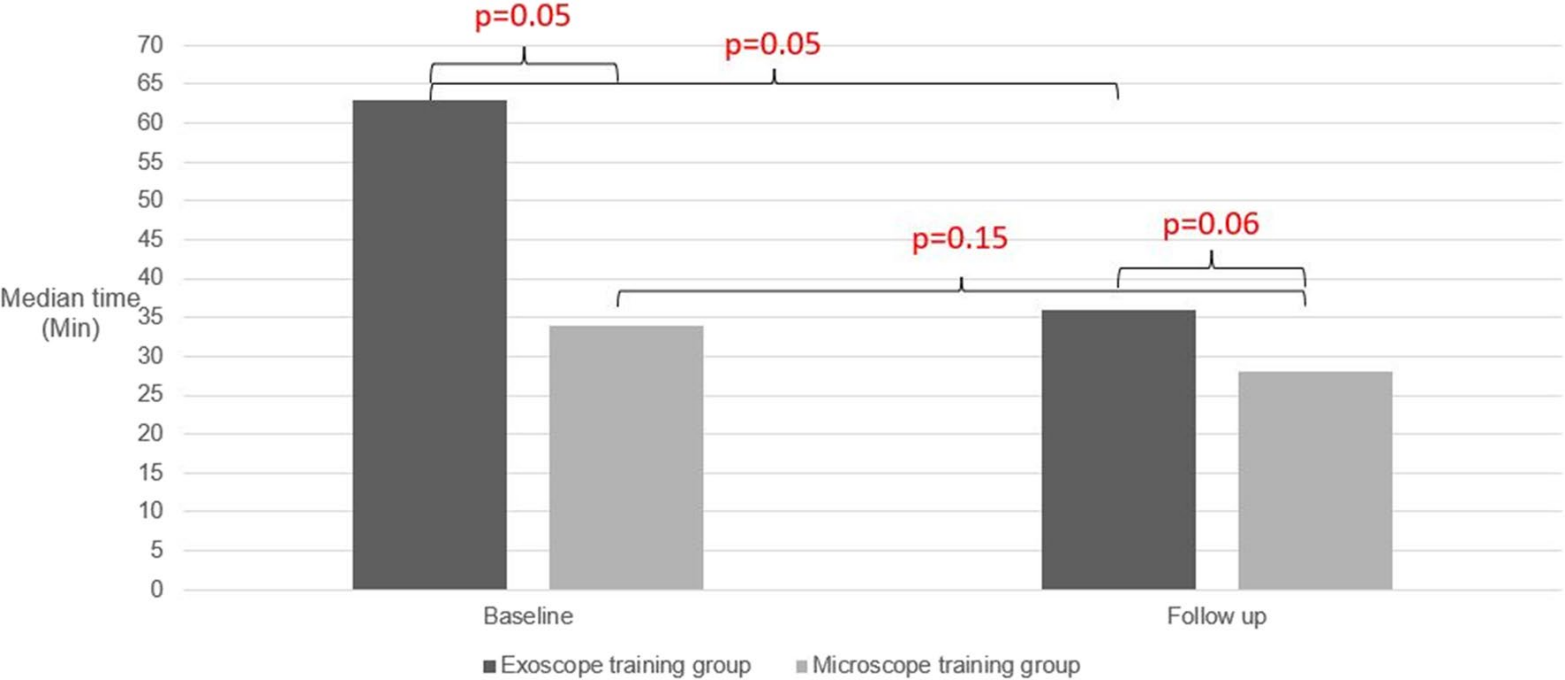
Duration of exoscope-assisted end-to-side anastomosis task among the participants trained with exoscope and microscope

At the follow-up, the median time shortened by 28 min (44%) in the exoscope training group and 6 min (17%) in the microscope training group (Table 1**; **Fig. 2). The reduction was steeper among the participants with less previous exoscope experience compared to the ones with more previous exoscope experience. One participant in the exoscope training group and one in the microscope training group did not improve their task duration. Both of them were experienced microsurgeons with previous exoscope experience.

**Table 1:** Exoscope-assisted end-to-side bypass tasks among the participants training with the exoscope and the microscope

	Exoscope training (*n* = 6)			Microscope training (*n* = 4)
	< 6 y experience (*n* = 2)	≥ 6 y experience (*n* = 4)	Total (*n* = 6)	
**Participants**				
Years of microsurgical training (median, (range))	2 (0.5–3)	7 (6–9)	6 (0.5–9)	4 (0.5–10)
Number of consultant neurosurgeons	0	4 (100%)	4 (67%)	2 (50%)
**Baseline**				
12 sutures completed	2/2 (100%)	4/4 (100%)	6/6 (100%)	4/4 (100%)
Duration of task^a^ Median (range)	64 min 16 s (3785–3927 s)	52 min 48 s (2331–4384 s)	64 min 16 s (2331–4384 s)	34 min 19 s (1747–3319 s)
Microsurgical dexterity–number of times per task (median (range)) when^b^: -Instruments out of field^c^ -Accidentally needle drop -Out of focus^d^	22 (21–23) 13 (13–13) 2 (0–4)	14 (2–26) 4 (2–13) 2 (1–7)	22 (2–26) 9 (2–13) 2 (0–7)	18 (6–30) 2 (1–8) 4 (0–6)
Quality of the anastomosis: -Equal suture distribution -Anastomosis orifice^e^	0/2 (0%) 1/2 (50%)	0/4 (0%) 3/4 (75%)	0/6 (0%) 4/6 (67%)	0/6 (0%) 2/4 (50%)
**Follow-up**				
12 sutures completed	2/2 (100%)	4/4 (100%)	6/6 (100%)	4/4 (100%)
Duration of task^a^ Median (range)	35 min 14 s (2034–2193 s)	40 min 9 s (1854–2887 s)	36 min 12 s (1854–2887 s)	28 min 26 s (1037–2123 s)
Microsurgical dexterity–number of times per task (median (range)) when^f^: -Instruments out of field -Accidentally needle drop -Out of focus	21 (12–30) 5 (3–6) 4 (3–4)	12 (4–20) 5 (4–5) 2 (1–4)	12 (4–30) 5 (3–6) 3 (1–4)	13 (2–24) 1 (0–3) 1 (1–2)
Quality of the anastomosis: -Equal suture distribution -Anastomosis orifice	1/2 (50%) 2/2 (100%)	0/4 (0%) 4/4 (100%)	1/6 (17%) 6/6 (100%)	1/4 (25%) 3/4 (75%)

^a^Time from applying the first temporary clip to recipient to removal of the last temporary clip after performing the anastomosis, also referred as ischemia time

^b^The recordings of performing the bypass were not available on one participant of the microscope group

^c^The both instruments are out of operative field

^d^The exoscope is out of focus

^e^The anastomosis orifice larger than the recipient caliber

^f^The recordings of performing the bypass were not available on one participant of the exoscope group

### Microsurgical dexterity and anastomosis quality

For the manual dexterity, both instruments were less frequently out of the surgical field (45% decrease) and the needle was dropped less often (44% decrease) during the follow-up task than during the baseline task in the exoscope training group. In regard to anastomosis quality, a wider anastomosis orifice was achieved in 33% more cases during the follow-up compared to the baseline. Equal suture distribution was the most difficult parameter to achieve. Only one out of six (17%) participants reached this goal in the exoscope group. The results of microsurgical dexterity and anastomosis quality are presented in Table 1. Overall, apart from two experienced participants, all the rest showed improvement in their microsurgical dexterity and suturing quality between the baseline and the follow-up task irrespective of whether they trained with the exoscope or the microscope.

### Previous microneurosurgical experience in the exoscope training group

The previous microsurgical experience affected the result in the exoscope training group, especially during the baseline task (Table 1). The participants with less than 6 years of microsurgical experience were slower than those with more than 6 years of experience (median 64 min *vs* 53 min). The less experienced participants struggled with both the microsurgical dexterity (more needle drops and instruments more frequently out of the field) as well as the anastomosis quality (orifice narrower than the recipient diameter).

**Fig. 3:**
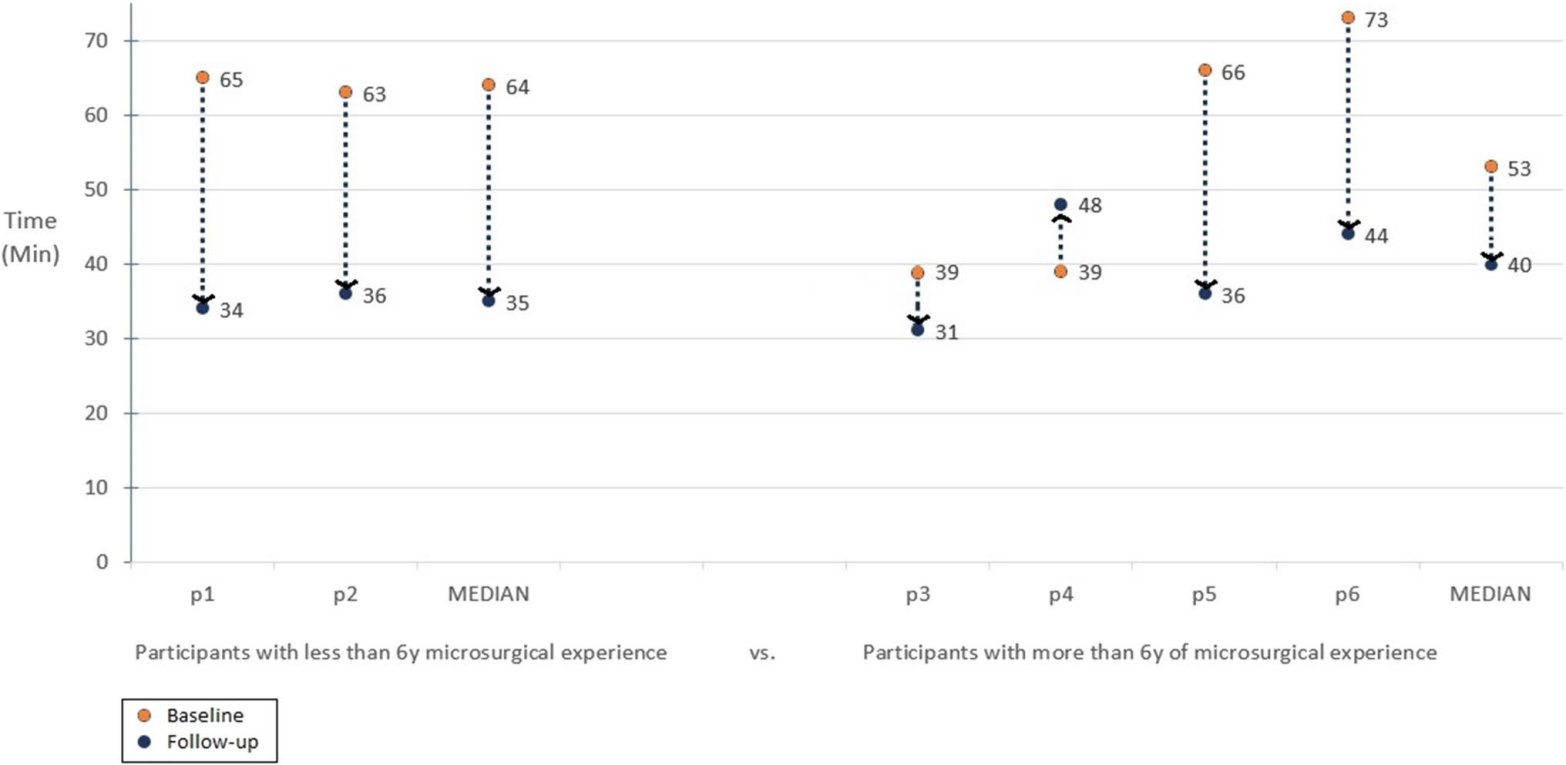
The change in the duration of the exoscope-assisted end-to-side anastomosis task among the participants training with exoscope

However, the less experienced participants had a much steeper learning curve during the six training sessions. In the follow-up task, they improved their time by a median of 29 min (45%) compared to the median of 13 min (24%) of the more experienced participants (Fig. 3). A participant with more than 6 years of experience did not improve his result at all. A steep reduction in accidental needle drops was observed only among the less experienced participants.

### Previous exoscopic experience in the exoscope training group

At the baseline task, the participants with less than 1 year of exoscopic experience (*n* = 4) performed slower than their more experienced (*n* = 2) peers (median 66 min *vs* 39 min, respectively).

On the follow-up, the less experienced exoscope users showed significant improvement in their suturing time but the experienced users stayed at the same level (30 min *vs* 1 min of improvement). A similar trend was observed also for the microsurgical dexterity as well as for the anastomosis quality. Less experienced users improved their performance, but the more experienced ones remained on a relatively stable level.

### Effect of training and adaptation to the test task

Both the test group (exoscope training) and the control group (microscope training) showed faster completion times at the follow-up task compared to baseline (median improvement 28 min vs 6 min). The most significant improvement was seen in less experienced participants training with the exoscope (45%), whereas only little improvement was observed in more experienced participants training with the microscope (24%). Especially, the experienced participants with more than 1 year of exoscope experience were not able to improve their speed in performing the task. The differences were not as coherent when considering the quality of the anastomosis or the microsurgical dexterity.

## Discussion

Supporting our first hypothesis, a steeper learning curve of exoscope-assisted microvascular anastomosis was observed among the less experienced participants. Only the more experienced participants with at least 1-year experience of using the exoscope did not improve their task duration. However, it should be noted that the more experienced participants took less time to perform the anastomosis at baseline. Due to this, improving the operation time was more challenging for them. The results of the study suggest that a relatively short training period can lead to a major development in exoscope-assisted surgery among neurosurgeons with limited microsurgical experience. Therefore, even short-term exoscope training (6 h during 2–5 weeks) in a laboratory may be enough for the initial adaptation required for operating on clinical cases. A further study would be needed to evaluate the optimal length of a short-term practice period. We believe that even experienced microsurgeons need to familiarize themselves with the use of exoscope in laboratory conditions prior to operating on the first patient. However, as our results showed, once the basic level is reached, further advances probably require much more practice time and repetition.

Supporting our second and third hypothesis, training with an exoscope was more effective than training with a microscope when the aim was to improve exoscopic microsurgical performance. Of note, also the microscope group improved, which may be due to adaptation to the bypass task and the material used. This suggests that a reference group should be used when studying microsurgical learning curves with different devices to minimize the effect of adaptation during data collection. In addition, the result underlines that microsurgical training is beneficial, regardless of the magnification device.

Supporting our fourth hypothesis, training-induced development was not observed among the most experienced participants. After a certain level of expertise, long-term training is necessary to gain any further improvement. There is a limit to how much one can improve during a short-term training period.

Previous studies on learning mechanisms in exoscope-assisted microsurgery are scarce [8, 10]. Few studies have reported relatively quick adaptation to using the exoscope in a laboratory setting [8]. In a study on microanastomosis suturing with latex vessel models (participants *n* = 6) using a 3D exoscope, the authors reported besides the positive features of exoscope (e.g., image quality, magnification, and illumination), improved overall efficiency after only five sessions [8]. A single surgeon laboratory study comparing 100 exoscopic to 100 microscopic microanastomoses showed that both of the magnification devices were effective for microanastomosis suturing, but the distribution of sutures was better with the exoscope [2]. However, even after a long training period, the microscopic anastomosis took less to finish. Similar result was observed in another single surgeon laboratory study [1]. Both authors suspected somewhat worse depth-perception with exoscope compared to microscope. On the other hand, previous studies have also confirmed the advantages of using the exoscope instead of the microscope (e.g., improved ergonomics, higher magnification, and shorter inspection time after aneurysm clipping), in both laboratory and clinical conditions [2, 3, 5, 9, 11].

### Strengths

Among the major strengths of the present study were the control group, challenging test task, standardized test model, and different level participants. The presence of a reference group training with the microscope allowed us to monitor adaptation to the test task. Without the control group, this adaptation could have been easily misjudged as an effect of the exoscope training process. A sufficiently challenging microsurgical task, differing greatly from the participants’ daily operations, enabled improvement even among the more experienced participants. Use of a commercial and standardized end-to-side microanastomosis model, e.g., not including the anatomical variations of the chicken wing vessels, enabled the comparison of the time and quality of the anastomoses between the different participants and different time points. Furthermore, the inclusion of surgeons with different amounts of previous microsurgical experience, equally divided among the two training groups, allowed us to assess the importance of previous microsurgical expertise on the learning curve for exoscope-assisted surgery.

### Limitations

We acknowledge some study limitations. First, the anastomosis task was conducted under laboratory conditions, which is why the results cannot be directly applied to clinical practice. For example, the tasks were performed at 10 mm depth, which is shallower than in the authentic procedure. Secondly, the study only included ten participants, limiting statistical analysis and making it harder to match the two groups. For logistical reasons it is challenging to have large group of participants from a single institution. Once the exoscopes become more common, multicenter studies should be arranged in the future to confirm our primary results. Thirdly, due to production inconsistency, there were differences in the wall thickness and diameter of the anastomosis tubes’ wall. Especially, the mismatch between the donor and the recipient vessels makes it more challenging to perform the anastomosis. This is why part of the learning effect can be explained by the adaptation to the material of the bypass model. Fourth, superficial anastomosis is a relatively static procedure requiring only limited movement and tilting of the magnification device. Tilting of the camera head is one of the major advantages of the exoscope during surgery compared to the microscope. This feature was not utilized to its full potential in this study, so possible improvements for other procedure types cannot be directly extrapolated. Fifth, the number of surgeons with previous expertise of endoscopic neurosurgery was smaller in the microscope group. Previous endoscopic experience may be beneficial in the adaptation to the exoscope. Sixth, we did not control the use of exoscope in the surgeries that the study participants performed during the data collection. None of the surgeons involved in the study did bypass surgeries prior or during the data collection. The bypass task differed a lot from their daily surgeries. Seventh, there were major differences in the length of baseline task between the two groups. We tried to balance the groups based on the participants’ previous experience with exoscopes and microscopes. However, based on our results, it might be more reasonable to assign the participants into the groups based on their performances during the baseline task rather than their previous history.

## Conclusion

Even short-term training with the exoscope led to marked improvements in exoscope-assisted end-to-side bypass suturing among novice microneurosurgeons. For the more experienced participants, a plateau in the initial learning curve was reached quickly. Much longer-term effort would probably be required to achieve further improvement in this user group. When switching from the microscope to the exoscope even experienced microsurgeons benefit from some laboratory training prior to clinical use.

## Supplementary Information

Below is the link to the electronic supplementary material.

Supplementary file1 (MP4 495259 KB)

## Abbreviations and acronyms

IQR: Interquartile range
MCA: Middle cerebral artery
SD: Standard deviation
STA: Superficial temporal artery
